# Amplification of Mitochondrial Activity in the Healing Response Following Rotator Cuff Tendon Injury

**DOI:** 10.1038/s41598-018-35391-7

**Published:** 2018-11-19

**Authors:** Finosh G. Thankam, Isaiah S. Chandra, Anuradha N. Kovilam, Connor G. Diaz, Benjamin T. Volberding, Matthew F. Dilisio, Mohamed M. Radwan, R. Michael Gross, Devendra K. Agrawal

**Affiliations:** Departments of Clinical & Translational Science and Orthopedic Surgery, Creighton University School of Medicine, Omaha, NE 68178 USA

## Abstract

Mitochondrial function following rotator cuff tendon injury (RCI) influences the tendon healing. We examined the mitochondrial morphology and function under hypoxia in the shoulder tendon tissue from surgically-induced tenotomy-RCI rat model and cultured swine tenocytes. The tendon tissue was collected post-injury on 3–5 (Group-A), 10–12 (Group-B), and 22–24 (Group-C), days and the corresponding contralateral tendons were used as control for each group. There was higher protein expression of citrate synthase *(P* < *0.0001)* [10.22 MFI (mean fluorescent intensity)] and complex-1 *(P* = *0.0008)* (7.86 MFI) in Group-A and Group-B that decreased in Group-C [*(P* = *0.0201)* (5.78 MFI and *(P* = *0.7915)* (2.32 MFI), respectively] compared to control tendons. The ratio of BAX:Bcl2 (Bcl2 associated x protein:B cell lymphoma 2) in RCI tendons increased by 50.5% (Group-A) and 68.4% (Group-B) and decreased by 25.8% (Group-C) compared to normoxic controls. Hypoxia increased β-tubulin expression *(P* = *0067)* and reduced PGC1-α *(P* = *0412)* expression in the isolated swine tenocytes with no effect on the protein expression of Complex-1 *(P* = *7409)* and citrate synthase *(P* = *0.3290)*. Also, the hypoxic tenocytes exhibited about 4-fold increase in mitochondrial superoxide *(P* < *0.0001)*, altered morphology and mitochondrial pore integrity, and increase in mitochondrial density compared to normoxic controls. These findings suggest the critical role of mitochondria in the RCI healing response.

## Introduction

Biochemical changes in the tendon matrix and tenocyte dysfunction are considered to be the initial events associated with rotator cuff tendon injury (RCI)^1^. Normally, the tenocytes are believed to undergo oxidative metabolism despite the hypovascular nature of the tendon tissue^2^. However, the adaptive mechanisms and mitochondrial energy metabolism which facilitate the tenocytes to survive under hypoxic conditions associated with RCI are largely unknown^1,2^. Moreover, the available data from histomorphological features of the tendon tissue are insufficient to draw meaningful information regarding the mitochondrial status, the degree of tendon damage and altered tendon phenotype, following the RCI^3^. The increased susceptibility of tenocytes to undergo the intrinsic pathway of apoptosis correlates with the impact of mitochondria in the pathology of RCI^4^. Also, there are limited reports on the health/functional status of mitochondria in the tenocytes following RCI. Hence, further investigation on the involvement of mitochondrial dysfunction in tenocytes could reveal the underlying molecular pathology of RCI and identify targets for intervention.

Even though tendon tissue withstands transient hypoxia, uninterrupted oxygen supply is essential for proper mitochondrial function^5,6^. Moreover, the ‘critical zone’ of enthesis is poorly vascularized and is the common anatomical site for majority of RCI suggesting an association of hypoxia with RCI^7^. In contrast, as the tendon matures there occurs a shift from oxidative phosphorylation towards anaerobic glycolysis^8^. However, during the injury and repair process the tenocytes switch back to oxidative metabolism, since the metabolic demand increases drastically following the RCI^8^. The disturbances in oxygen homeostasis, as a result of hypoxia, trigger the expression of hypoxia inducible factor–1alpha (HIF-1α) which signals to elicit mitochondrial dysfunction and inflammation^7,9,10^. The RCI-tendons display an upregulation of HIF-1α and the downstream mediators suggesting hypoxia to be the major contributing factor in RCI^11,6^.

The increasing demand for cellular energy in tenocytes during RCI is believed to trigger mitochondrial biogenesis^12^. The key regulator for mitochondrial biogenesis, peroxisome proliferator-activated receptor gamma coactivator-1 alpha (PGC-1α), has been reported to be constantly higher in the diseased and stressed cells^13^. The interaction of PGC-1α with nuclear respiratory factor-1 (NRF-1) activates the mitochondrial transcription factor A (Tfam) to enhance the mitochondrial gene expression^13^. In addition, the increased cellular demand for metabolites and ATP deprivation following the RCI results in the increased levels of AMP, inorganic phosphate (Pi), divalent calcium ions and reactive oxygen species (ROS) which result in cellular stress^14^. ROS and oxidative stress are potent risk factors for RCI. Also, PGC-1α elicits antioxidant response by inducing the superoxide dismutase, catalase and peroxidases^15,16^. Since mitochondria are actively involved in oxidative metabolism, the decreased oxygen availability of tissues following an injury influences the mitochondrial activity^17,18^. However, the association of mitochondrial function and activity and the effect of hypoxia in the shoulder tendon tissues following RCI have not yet been studied. Therefore, we hypothesize that the health status of mitochondria under the hypoxic environment following acute RCI is strongly associated with the severity and delayed healing responses. Also, the tenocyte in culture responds to hypoxic conditions by increasing the mitochondrial activity. The present study aims at the investigation of mitochondrial health status in the tendon tissue of tenotomy-RCI-rat model and cultured swine tenocytes under hypoxic conditions.

## Results

Tenotomy was surgically induced in rat shoulder tendon to develop the RCI model and the injured tendon showed a visible difference in appearance when compared with the contralateral shoulder tendon as the control^19^. Histological examination of the RCI tendons displayed a drastic increase in cell density, matrix disorganization, inflammation, and fatty infiltration, which are the hallmarks of RCI pathology. We recently reported that the disorganized histologic structure matured as the healing occurred with more normal appearance of the tendon on 22–24 days following injury compared to the tendon on earlier time points^19^.

### Protein expression of mitochondrial biomarkers in RCI-rat shoulder tendon

**Figure 1:**
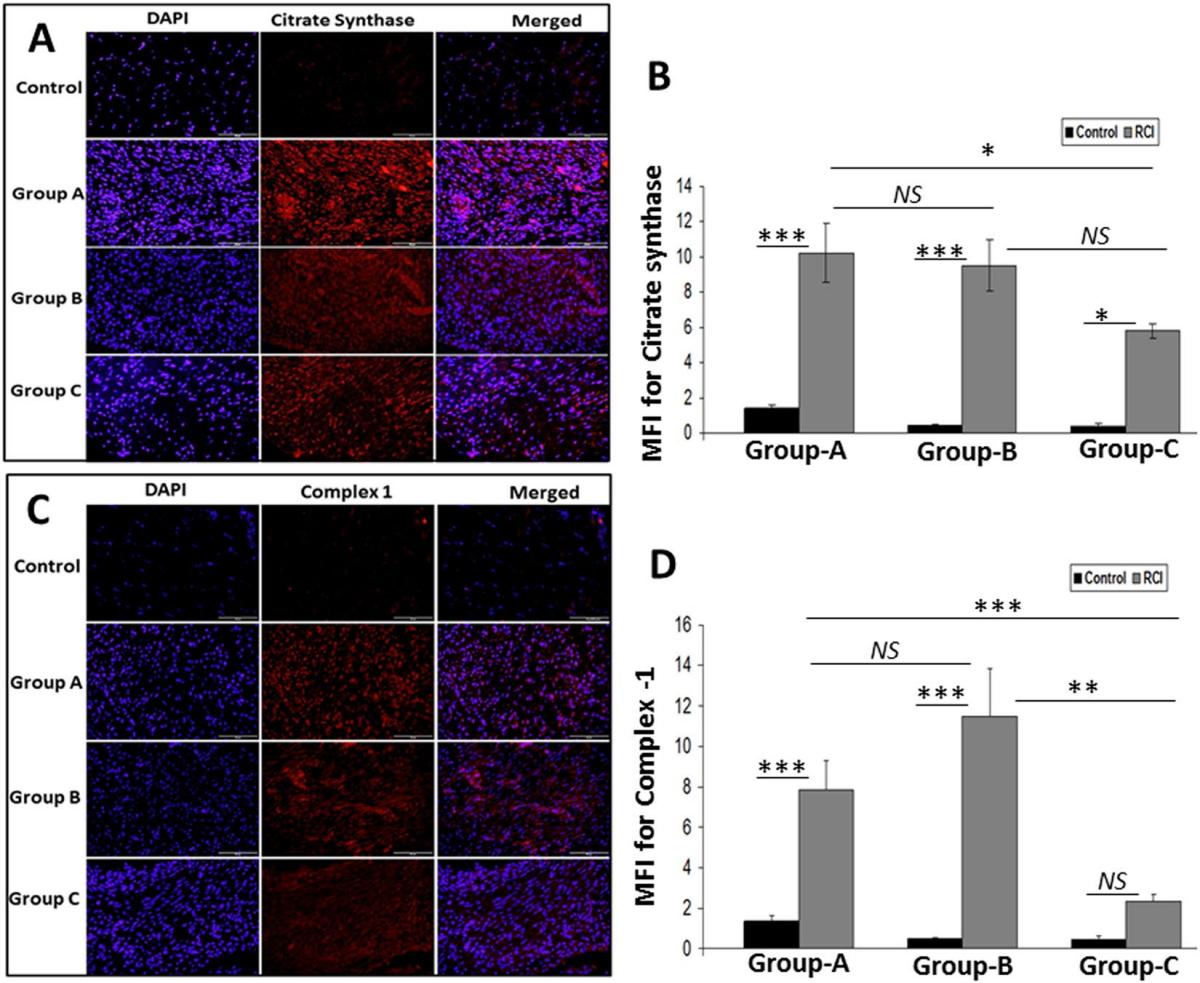
Immunofluorescence analysis for the expression of (**A**) Citrate synthase and (**C**) Complex-1 showing increased expression in RCI group on comparison with control. Images in the top row are histological sections of control group, and images in the bottom rows are histological sections of RCI tendons harvested at 3–5 days (Group-A), 10–12 days (Group-B) and 22–24 days (Group-C). Images in the left column show nuclear staining with DAPI; the images in the middle column show expression of Citrate synthase and Complex-1 while the images in the right column show overlay of Citrate synthase/Complex-1staining with DAPI. Images were acquired at 20x magnification using CCD camera attached to the Olympus microscope. (**B**,**D**) The image shows quantification of protein expression. The intensity of protein expression as observed through immunofluorescence was acquired and the mean fluorescence intensity (MFI) was quantified from each contralateral control and RCI specimen. The graphs represent MFI mean values with standard error. The statistical significance of each contralateral control groups *vs* RCI groups and between RCI groups are represented in the figure (n = 7; NS – non-significant, *P < 0.05, **P < 0.01 and ***P < *0.001*) and the P values for RCI specimen within and between the groups are displayed in Supplementary Table 1.

**Figure 2:**
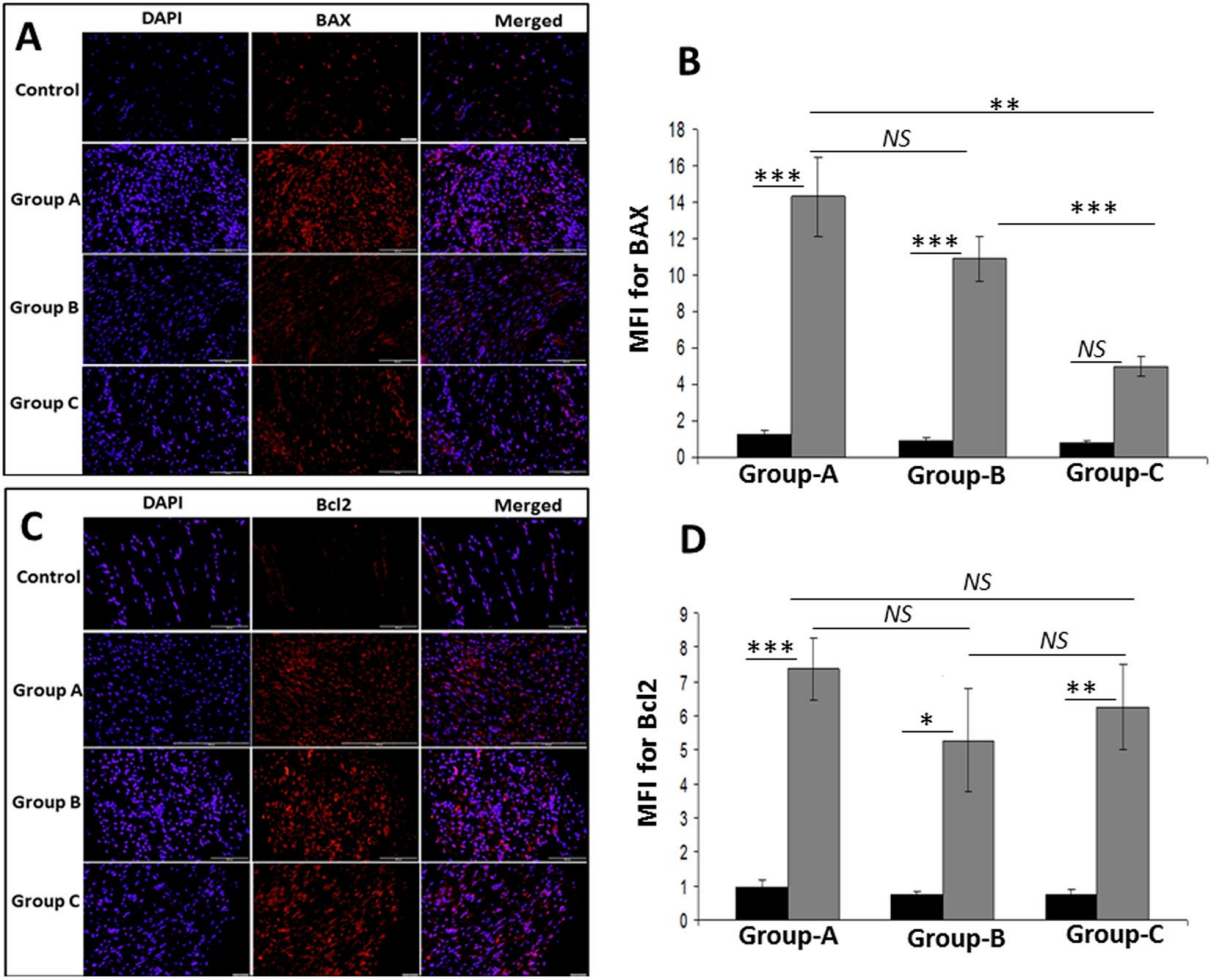
Immunofluorescence analysis for the expression of (**A**) BAX and (**C**) Bcl2 showing increased expression in RCI group on comparison with control. Images in the top row are histological sections of control group, and images in the bottom rows are histological sections of RCI tendons harvested at 3–5 days (Group-A), 10–12 days (Group-B) and 22–24 days (Group-C). Images in the left column show nuclear staining with DAPI; the images in the middle column show expression of BAX/Bcl2 while the images in the right column show overlay of BAX/Bcl2 staining with DAPI. Images were acquired at 20x magnification using CCD camera attached to the Olympus microscope. (**B**,**D**) The image shows quantification of the expression of BAX and Bcl2. The intensity of protein expression as observed through immunofluorescence was acquired and the mean fluorescence intensity (MFI) was quantified from each contralateral control and RCI specimen. The graphs represent MFI mean values with standard error. The statistical significance of each contralateral control groups *vs* RCI groups and between RCI groups are represented in the figure (n = 7; NS – non-significant, *P < 0.05, **P < 0.01 and ***P < *0.001*) and the P values for RCI specimen within and between the groups are displayed in Supplementary Table 1.

The protein expression of mitochondrial biomarker citrate synthase was significantly increased in RCI tendons when compared with corresponding controls in Group-A (10.22 ± 1.62 MFI), Group-B (9.51 ± 1.44 MFI units) and Group-C (5.78 ± 0.45 MFI units) (*P* < *0.0001, P* < *0.0001* and *P* = *0.0201*, respectively). The expression of synthase did not change significantly between Group-A and Group-B RCI tendons (*P* = *0.9952*), however was decreased non-significantly in Group-C RCI tendons than Group-B (*P* = *0.1583*) and significantly decreased compared to Group-A (*P* = *0.0469*). Levels of citrate synthase did not change significantly over time in the control tendons (Fig. 1A,B). Similarly, the protein expression of mitochondrial protein complex-1 was significantly increased in the RCI tendons compared to corresponding controls in Group A (7.86 ± 1.42 MFI units) and Group B (11.47 ± 2.38 MFI units) (*P* = *0.0008* and *P* = <*0.0001*, respectively). Complex-1 was also increased in Group-C RCI tendons compared to control, though this result was not statistically significant (p = 0.7915). The expression of complex-1 did not change significantly between Group-A and Group-B RCI tendons (*P* = *0.2649*), however significantly decreased in Group-C RCI tendons when compared with Group-A and Group-B (*P* = *0.0075 and P* < *0.0001*, respectively). Levels of complex-1 did not change significantly over time in the control tendons. In RCI tendons, complex-1 expression was significantly less in Group-C (2.32 ± 0.34 MFI units) compared to both Group-A (p = 0.0075) and Group-B (<0.0001) (Fig. 1C,D). The pro-apoptotic protein BAX was significantly higher in both RCI-Group-A *(P* < *0.0001)* (14.3 ± 2.15 MFI units) and RCI-Group-B *(P* < *0.0001)* (10.91 ± 1.22 MFI units) compared to the corresponding controls and was also higher in Group-C RCI tendons compared to control, though this result was not statistically significant (p = 0.0704). The expression of BAX did not change significantly between Group-A and Group-B RCI tendons (*P* = *0.2556*), however significantly decreased in Group-C RCI tendons when compared with Group-A and Group-B (*P* < *0.0001 and P* = *0.0056*, respectively). The BAX expression was less in all control tendons, though this result was not statistically significant (Fig. 2A,B). The anti-apoptotic protein Bcl2 in RCI tendon was higher in Group-A (7.36 ± 0.93 MFI units), Group-B (5.27 ± 1.51 MFI units), and Group-C (6.25 ± 1.24 MFI units), however the result was not statistically significant in Group-B when compared with Group-A and Group-C (*P* = *0.5942* and *P* = *0.9762*, respectively) (Fig. 2C,D). The Bcl2 levels were significantly higher in RCI groups when compared with the control tendons (*P* = *0.0001, P* = *0.016* and *P* = *0.0012*, respectively). Basal levels of Bcl2 protein expression were observed in all three control groups (Fig. 2A,C, top row), however, was statistically not significant. The BAX: Bcl2 ratio was calculated from the average MFI from each group (Table 1). The *P* values and statistical significance within and between the groups for the mitochondrial biomarkers (citrate synthase, complex 1, BAX and Bcl2) were calculated using two-way ANOVA, and are displayed in Supplementary Table 1.

**Table 1:** BAX: Bcl2 ratio and the % change compared to contralateral control. The ratio is calculated from the average MFI from each group.

Groups	Control	RCI	% Change
Group-A	1.29	1.94	50.51% increase
Group-B	1.23	2.07	68.44% increase
Group-C	1.08	0.80	25.82% decrease

### Characterization of swine tenocytes

**Figure 3:**
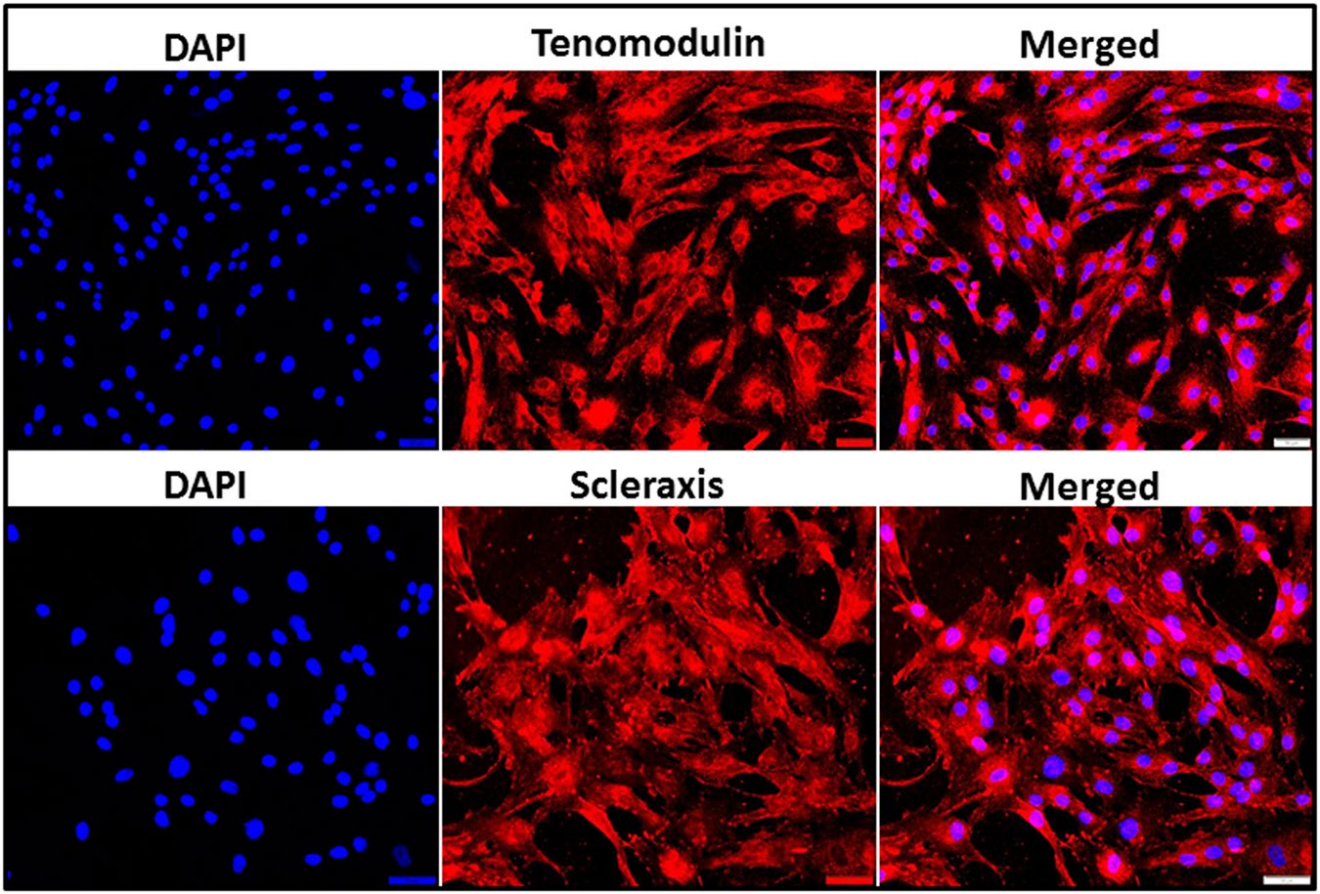
Characterization of tenocytes: Immunofluorescence analysis for the protein expression of (**A**) Tenomodulin and (**B**) Scleraxis showing their expression. Images in the left column show nuclear staining with DAPI; the images in the middle column show expression of Tenomodulin and Scleraxis while the images in the right column show overlay of Tenomodulin and Scleraxis staining with DAPI. Images were acquired at 20x magnification using CCD camera attached to the Olympus microscope.

The tenocytes were isolated from the infraspinatus tendon of Yucatan micro-swine by explant culture method following partial collagenase digestion. The protein expression of tendon specific biomarkers, including tenomodulin and scleraxis, characterized the isolated cells as tenocytes (Fig. 3).

### Expression of mitochondrial markers in swine tenocytes under hypoxia

**Figure 4:**
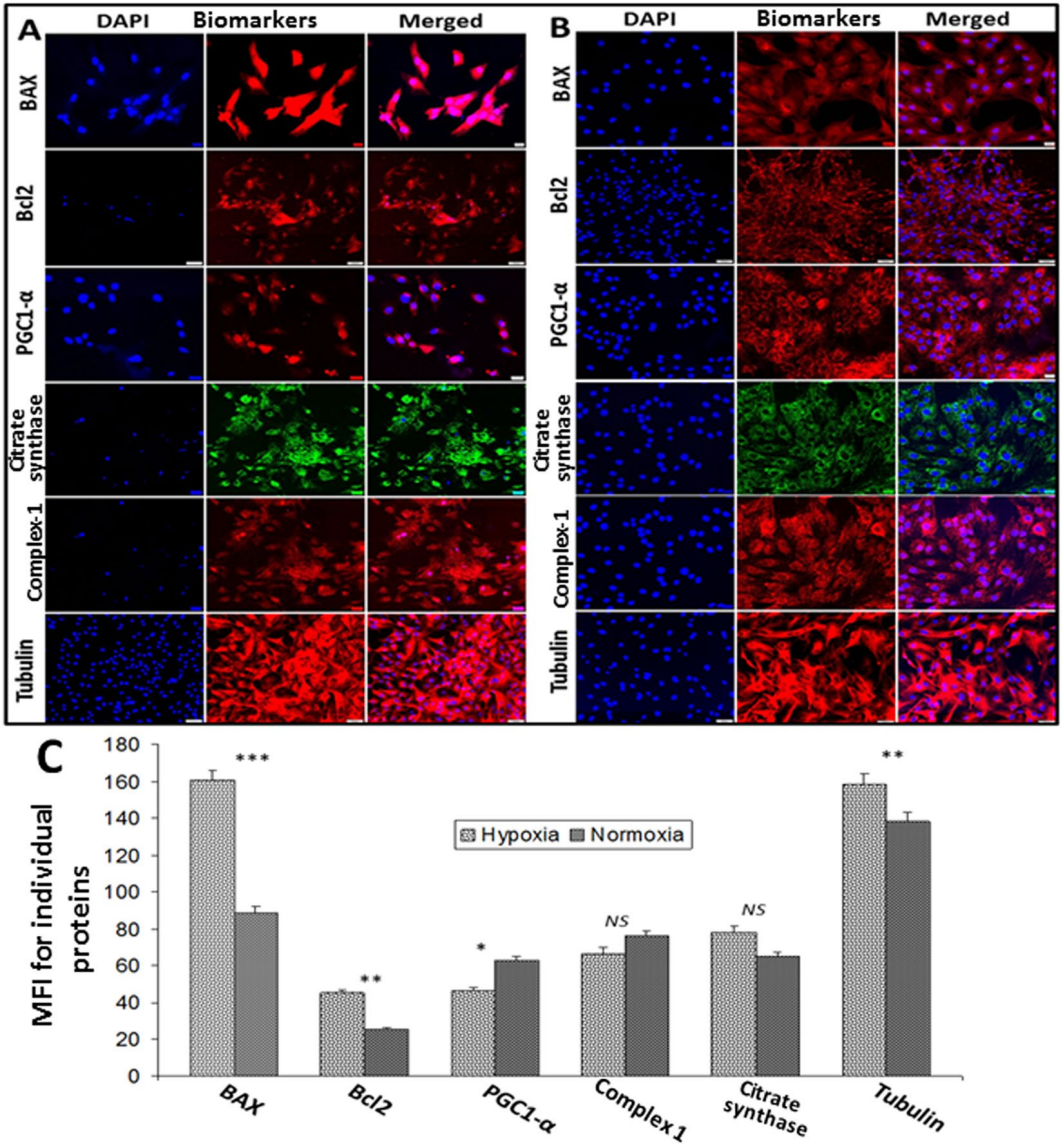
Immunofluorescence analysis for the protein expression of mitochondrial biomarkers by tenocytes cultured under (**A**) hypoxic and (**B**) normoxic conditions: Images in the left columns show nuclear staining with DAPI; the images in the middle column show expression of mitochondrial biomarkers while the images in the right column show overlay of mitochondrial biomarkers with DAPI. Images were acquired at 20x magnification using CCD camera attached to the Olympus microscope. (**C**) The image shows quantification of the expression mitochondrial biomarkers. The intensity of protein expression as observed through immunofluorescence was acquired and the mean fluorescence intensity (MFI) was quantified from 30 cells of each groups. The graphs represent MFI mean values with standard error. The statistical significance of each hypoxic groups *vs* normoxic groups are represented in the figure (n = 30; NS – non-significant, *P < 0.05, **P < 0.01 and ***P < *0.001*).

The isolated swine tenocytes were subjected to hypoxia (2% O_2_) and the protein expression of apoptotic and mitochondrial biomarkers were examined. The pro-apoptotic biomarker BAX was significantly higher (82.20% increase, *P* < *0.0001*) in hypoxic cells than normoxic controls. The protein expression of Bcl2, the anti-apoptotic biomarker, was also significantly higher (77.57% increase, *P* = *0.0054*) in hypoxic cells than normoxic cells. However, no considerable change in the ratio of BAX: Bcl2 (3.57 and 3.47, respectively for hypoxic and normoxic tenocytes) was evident when compared with hypoxic *vs* normoxic tenocytes (Fig. 4A,B). The protein expression of PGC1-α, the biomarker for mitochondrial biogenesis, was significantly lower (26.49% decrease, *P* = *0.0412*) in hypoxic tenocytes, whereas the level of mitochondrial proteins, such as Complex-1 (*P* = *7409*) and citrate synthase (*P* = *0.3290*), was not significantly varied between the groups (Fig. 4A,B). The protein expression of β-tubulin was greater (14.39% increase, *P* = *0067*) in hypoxic tenocytes when compared to normoxic controls (Fig. 4A,B).

### mRNA transcripts for mitochondrial markers under hypoxia

**Figure 5:**
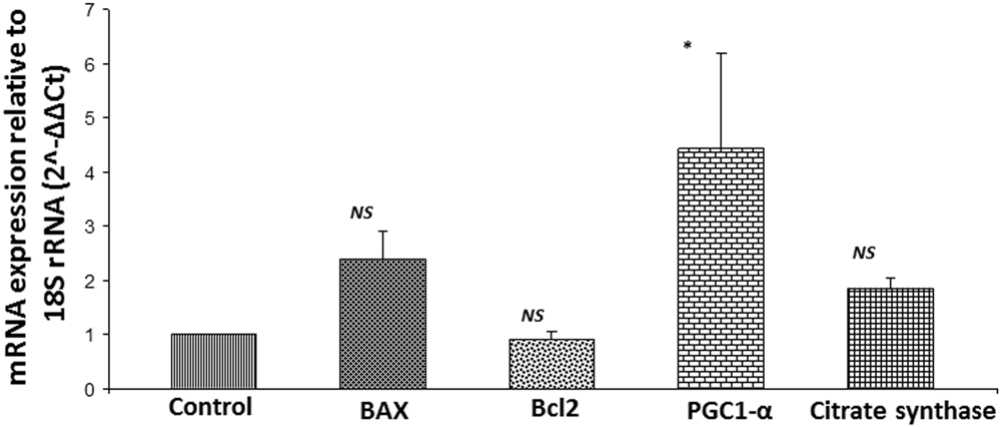
qRT-PCR analysis for the mRNA expression of mitochondrial biomarkers by tenocytes cultured under hypoxic with respect to normoxic controls: The results were expressed as fold change of expression relative to control. (n = 4; *NS – non-significant*, and *P < *0.05*).

The mRNA transcript levels of BAX and citrate synthase were found to be increased (2.38 fold and 1.86 fold, respectively) in hypoxic tenocytes when compared with normoxic cells; however the result was not statistically significant. Bcl2 expression was similar to that of the control. The ratio of BAX:Bcl2 mRNA transcript was found to be 2.64 in hypoxic tenocytes when compared to normoxic tenocytes. In addition, PGC1-α mRNA transcripts were significantly higher (4.44 fold increase, *P* = *0.0331*) in hypoxic group than normoxic control cells (Fig. 5).

### Determination of mitochondrial superoxide

**Figure 6:**
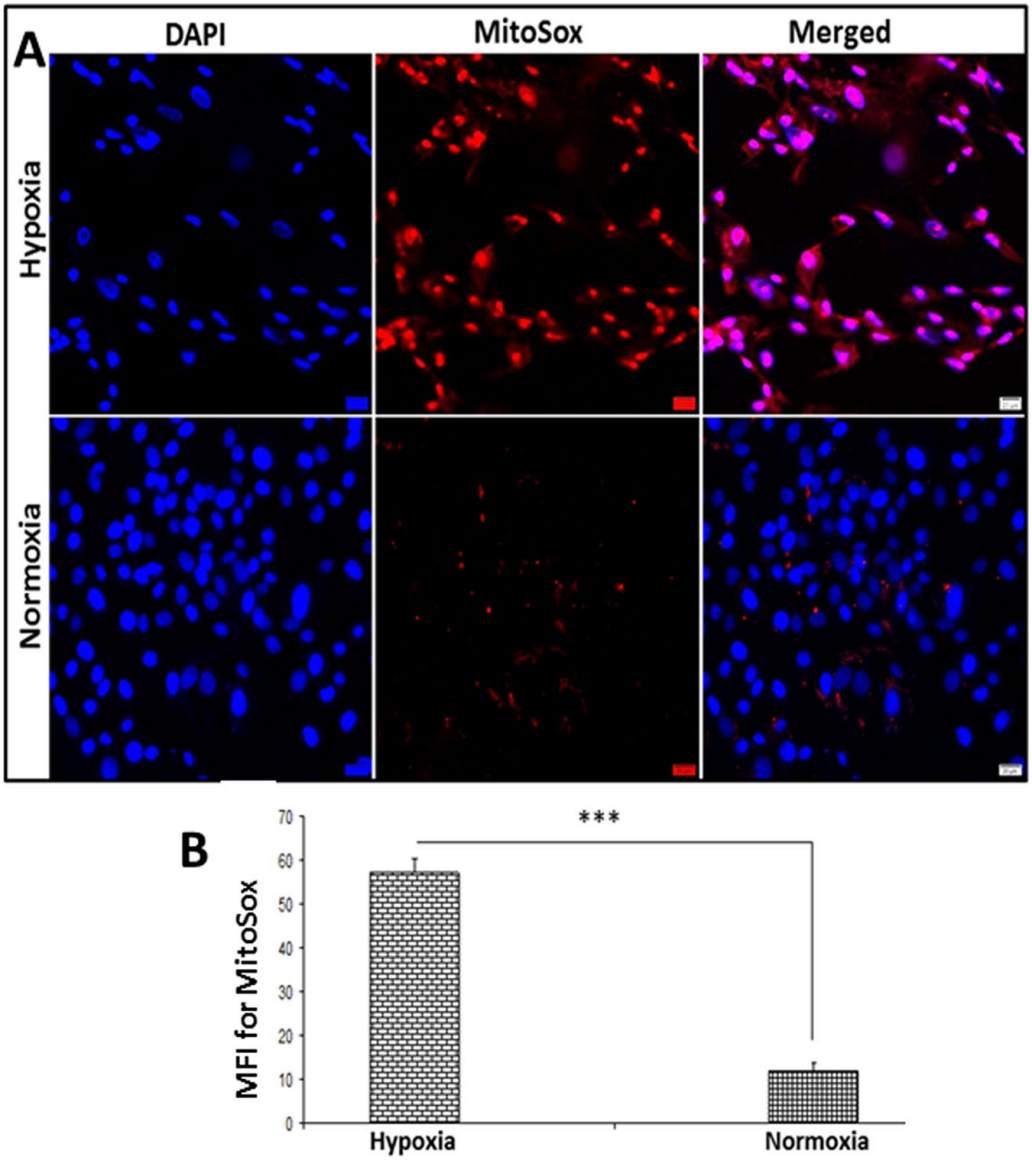
(**A**) Determination of mitochondrial superoxide using MitoSox showing increased superoxide in hypoxic tenocytes with normoxic control. Images in the left column show nuclear staining with DAPI; the images in the middle column show expression of superoxide while the images in the right column show overlay of MitoSox with DAPI. Images were acquired at 20x magnification using CCD camera attached to the Olympus microscope. (**B**) The image shows quantification of the mitochondrial superoxide. The intensity of protein expression as observed through immunofluorescence was acquired and the mean fluorescence intensity (MFI) was normalized to 100 cells. The graphs represent MFI mean values with standard error. The statistical significance of each hypoxic groups *vs* normoxic groups are represented in the figure (n = 4; NS – non-significant, *P < 0.05, **P < 0.01 and ***P < *0.001*).

There was an increase of 375.8% (*P* < *0.0001*) in mitochondrial superoxide in hypoxic tenocytes compared to the tenocytes grown under normoxic conditions as determined by MitoSox Red assay. However, basal level of expression was observed in normoxic tenocytes (Fig. 6A,B).

### Mitochondrial pore transition (MPT) assay

**Figure 7:**
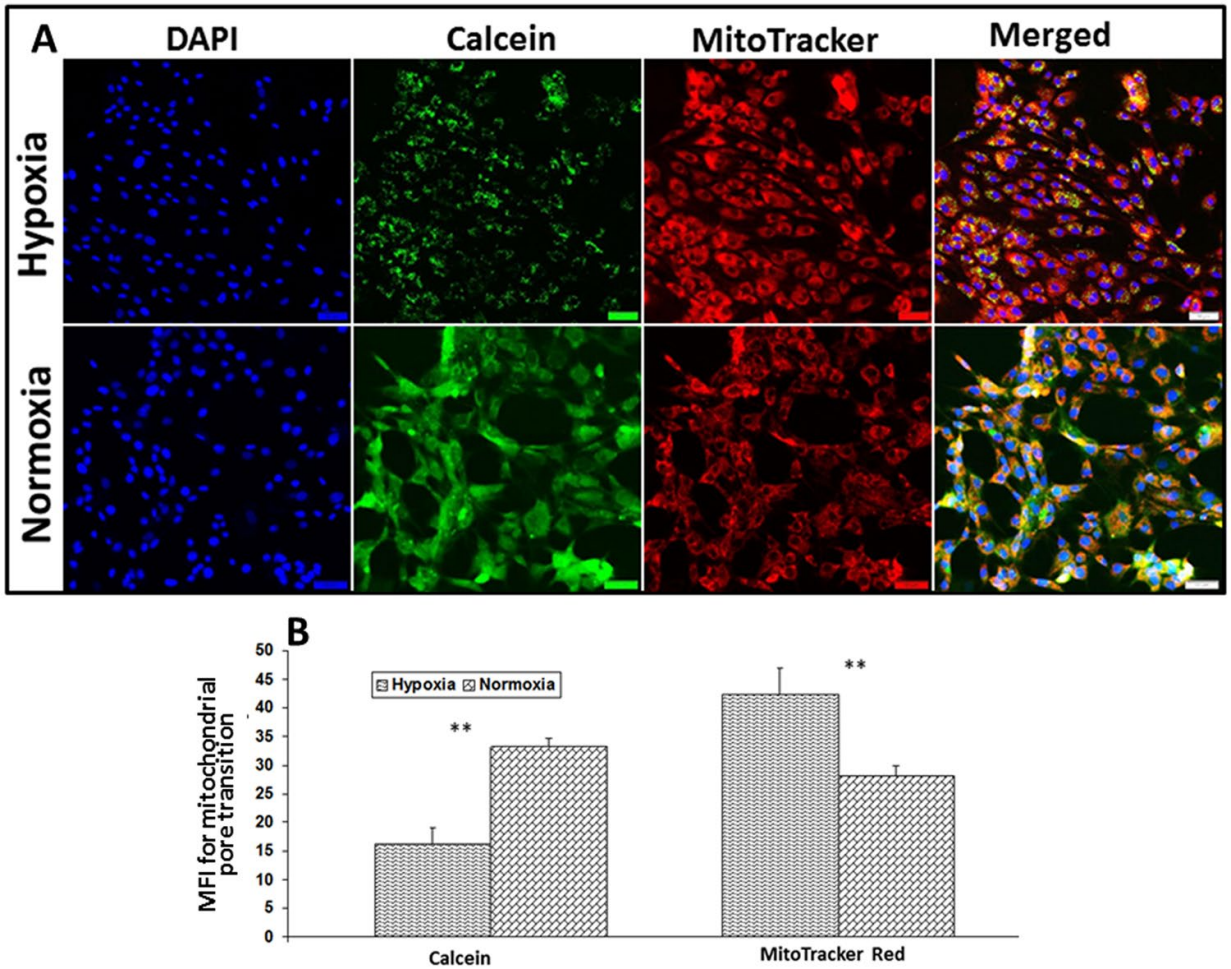
(**A**) Determination of mitochondrial pore transition using Image-iT LIVE mitochondrial transition pore assay Kit in hypoxic *vs* normoxic tenocytes. Images in the left column show nuclear staining with DAPI; the images in the middle columns show Calcein production and mitochondrial content while the images in the right column show overlay with DAPI. Images were acquired at 20x magnification using CCD camera attached to the Olympus microscope. (**B**) The image shows quantification of the mitochondrial pore transition. The intensity of protein expression as observed through immunofluorescence was acquired and the mean fluorescence intensity (MFI) was normalized to 100 cells. The graphs represent MFI mean values with standard error. The statistical significance of each hypoxic groups *vs* normoxic groups are represented in the figure (n = 4; NS – non-significant, *P < 0.05, **P < 0.01 and ***P < *0.001*).

The intensity of the calcein fluorescence was significantly higher (103.61% increase, *P* = *0.0032*) in normoxic tenocytes than the hypoxic cells (Fig. 7A,B). Since the healthy mitochondrial membrane retains more calcein resulting in higher fluorescence intensity due to the impermeability of CoCl_2,_ the findings suggest poor mitochondrial membrane integrity in hypoxic tenocytes. This could also be supported by the finding of the fluorescence with MitoTracker Red which was significantly higher in hypoxic cells than that of normoxic tenocytes. The average increase of MFI corresponding to MitoTracker Red in hypoxic cells was 50.8% (*P* = *0063*) greater than normoxic tenocytes which implies more mitochondrial density in hypoxic cells (Fig. 7A,B).

### Mitochondrial morphology by transmission electron microscopy (TEM)

**Figure 8:**
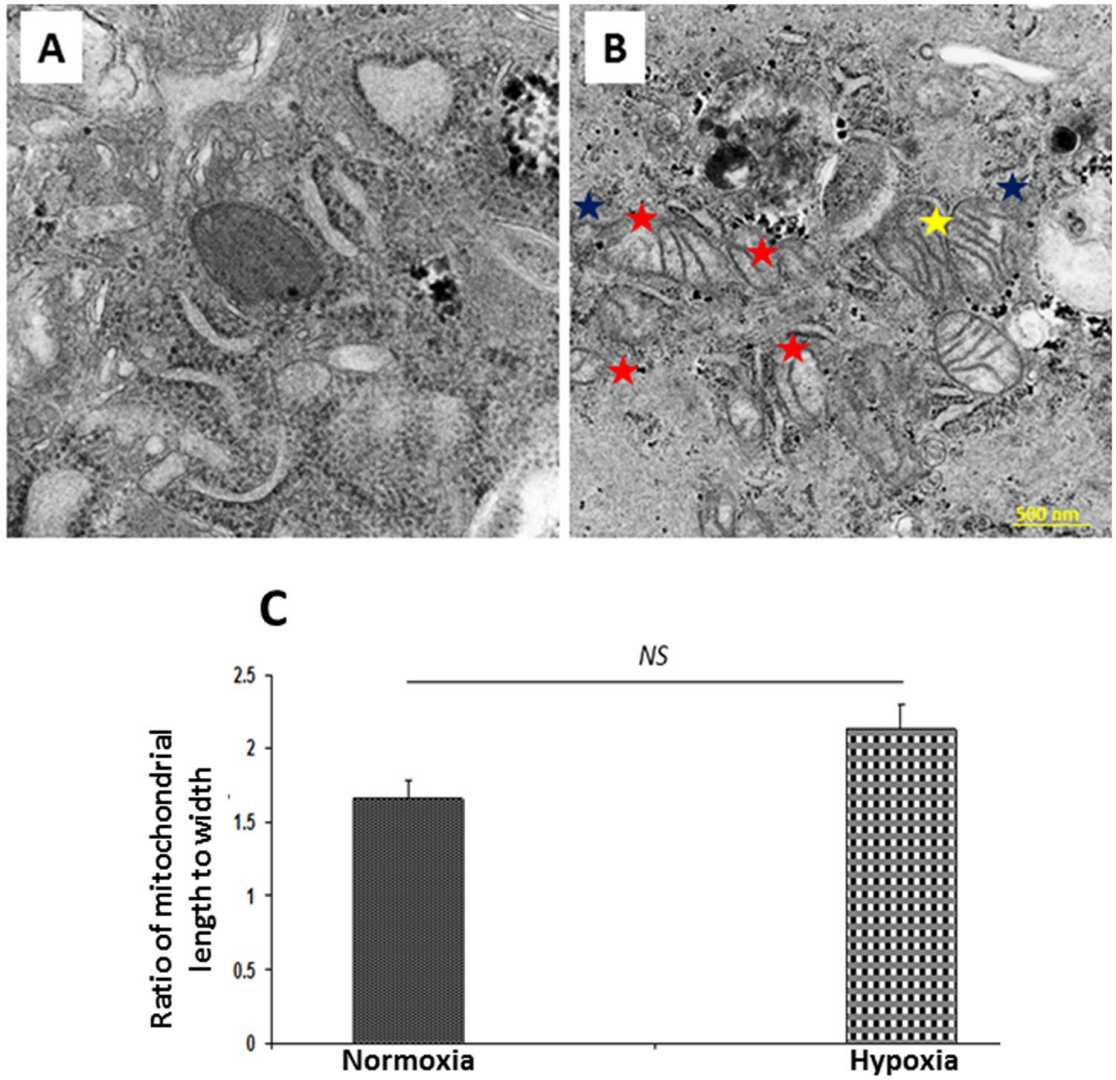
The examination of mitochondrial pathology using TEM analysis in tenocytes grown under (**A**) normoxic and (**B**) hypoxic conditions. Trans-mitochondrial cristae co-ordination (yellow star), mitochondrial compartmentalization (red star) and extension of outer mitochondrial membrane (blue star) were completely absent in normoxic cells when compared with hypoxic cells (**B**) and the normoxic (**A**) mitochondria exhibited higher electron density. (**C**) The figure shows non-significant increase in the mitochondrial aspect ratio in hypoxic mitochondria (n = 27) than that of normoxic ones (n = 10) (*NS – non-significant*).

TEM analysis revealed alterations in the characteristic morphological features of mitochondria between normoxic and hypoxic tenocytes (Fig. 8A,B). The qualitative analysis of TEM images revealed increased mitochondrial density in hypoxic tenocytes than the normoxic cells (Fig. 8A,B). Coordination of the trans-mitochondrial cristae was evident in hypoxic tenocytes which was absent in normoxic counterpart (Fig. 8A,B). The mitochondrial compartmentalization was prominent in hypoxic tenocytes as evident by the difference in electron densities between the compartments. The compartment formation was found to be an extension of the cristae membrane. However, the cristae junctions were completely absent (Fig. 8B). Also, the extension of outer mitochondrial membrane towards cytoplasm was evident in hypoxic cells (Fig. 8B). Coordination of the trans-mitochondrial cristae, mitochondrial compartmentalization and extension of outer mitochondrial membrane were completely absent in normoxic cells and the normoxic mitochondria were electron dense (Fig. 8A). The 28.35% increase in mitochondrial aspect ratio was observed in hypoxic mitochondria (n = 27) than that of normoxic ones (n = 10), however the increase was not statistically significant (*P* = *0.1531*) (Fig. 8C).

## Discussion

The anatomical architecture of rotator cuff tissue, especially the supraspinatus tendon in the subacromial space, makes it vulnerable to pathological changes^1^. The distal supraspinatus tendon is hypovascular and is highly susceptible to hypoxic insult, which in turn triggers a cascade of events resulting in RCI. Our tenotomy rat model displayed classical pathological features and abnormal changes in the tendon matrix^19^. Focus of this study was to investigate the role of mitochondrial function and activity in aggravating RCI pathology and healing responses. The tenotomy rat model mimics acute RCI pathology and is ideal for understanding the tendon self-healing mechanisms as the rats recover from tendon damage within a period of 4 months^19–21^. Moreover, the basic anatomical and pathophysiological mechanisms are considered to be similar to that in human^22^. In general, the injury limits the availability of oxygen to the tissues and leads to hypoxic stress which aggravates the pathology^18,23^. Similarly, the tenotomy induces hypoxic insult in tendon tissue and influences the mitochondrial function. Therefore, the tenotomy model is apt for understanding the underlying pathological events following acute RCI. Hypoxia has been considered to be the initial event associated with RCI which may result in increased mitochondrial biogenesis by activating PGC1-α^15,24,25^. Also, the increased mitochondrial density and activity have been associated with the early phases of healing response in several tissues^26^. In our recently reported findings in the RCI model, we observed an upregulation of PGC1-α in the tendon tissue on 3–5 days and 10–12 days following injury in the rotator cuff^19^. These findings could also be supported by the data in this study where the expression of mitochondrial biomarkers, such as citrate synthase and complex-1, was decreased in later phase of healing (Group-C). These results strongly support an association of mitochondria in the healing of tendon tissue following RCI.

The cultured tenocytes under hypoxia revealed a decrease in PGC1-α when compared with the normoxic cells. However, the level of citrate synthase and complex-1 were similar in both normoxic and hypoxic tenocytes. The contrasting results obtained from *in vivo* and *in vitro* examination of citrate synthase and complex-1 signify that the factors other than hypoxia contribute towards mitochondrial dysfunction in tendon tissues. For example, the proinflammatory cytokines including TNF-α, and IL-1β can influence the mitochondrial activity and function^27,28^. Following RCI, the level of proinflammatory cytokines, cellular apoptosis and immune cell infiltration in the rotator cuff tendon increase drastically^19^. In addition, danger-associated molecular patterns (DAMPs)-mediated sterile inflammation has been reported to be prevalent in tendon tissues which activates several inflammatory cascades^29^. The surviving tissue compromises the energy demand by channeling the metabolic flux for energy production by increasing the mitochondrial function. This could be the reason for the increased mitochondrial biomarkers in RCI tendons which progressively declined during healing process.

Moreover, the hypoxia is known to be an inducer of ROS generation *via* Complex III^30^ and a drastic increase in superoxide level was observed in the hypoxic tenocytes. In response to ROS, PGC1-α compromises the energy metabolism by increasing mitochondrial biogenesis^31^. However, the HIF1-α activation following extreme hypoxia inhibits PGC1-α^32,33^. Moreover, the balance between PGC1-α and ROS is necessary to sustain mitochondrial function in the surviving tendon tissues following RCI^31^. Under *in vivo* conditions, PGC1-α orchestrates the inflammatory response triggered by various DAMPs and pathogen associated molecular patterns (PAMPs) to facilitate mitochondrial biogenesis, especially by activating the pattern recognition receptors (PRRs), including toll-like receptors (TLRs)^34^. In addition, the increased demand for metabolic flux for tissue repair following the injury channels the mitochondrial biogenesis through the activation of PGC1-α^35^. This suggests that hypoxia alone is not a trigger for mitochondrial dysfunction following RCI and may explain the underlying mechanism of increased PGC1-α expression in the rat RCI tissue when compared to cultured hypoxic tenocytes.

Apoptosis of tendon cells has also been considered to be the hallmark for RCI pathology which involves mitochondrial pathways^36^. Even though the tenocytes are resistant to transient hypoxia, the persistent hypoxia drives apoptosis of the tenocytes following RCI in response to inflammatory cytokines^37^. Consequently, a disturbance in tendon matrix homeostasis due to the switch of collagen synthesis from Collagen I to Collagen III occurs which is responsible for the loss of mechanical integrity and subsequent tendon function^37^. Recently, we reported the impact of phenotype switch from collagen type I to type III in the biceps tendon tissues of human RCI patients^29,38^ and also in animal models^19^. However, further investigations are warranted on the relationship between mitochondrial dysfunction/apoptosis and alteration in collagen I to III ratio.

In the present study, the apoptotic biomarker BAX was found to be significantly higher in Group-A and Group-B and progressively decreased in Group-C in the RCI-rats. The expression of anti-apoptotic biomarker Bcl2 was higher in all three groups. Similar increase in BAX and Bcl2 expression were found in hypoxic tenocytes. Interestingly, the BAX: Bcl2 ratio was decreased as the healing progressed in the RCI-tendons of the rat model. Also, the ratio was similar in the control tendon groups of RCI rats and was unaltered between cultured hypoxic and normoxic tenocytes. The apoptotic cells survive by the upregulation of pro-survival mediators and anti-apoptotic proteins such as Bcl2^39^. This suggests that the tendon cells maintain the balance between BAX and Bcl2 especially in response to a damage/stress which was evident from the restoration of the ratio in the healing phase (Group-C).

The mRNA transcript levels of BAX revealed an increase similar to that in the BAX protein expression. The increased BAX:Bcl2 ratio suggests the mitochondrial dysfunction and susceptibility of tenocytes to apoptosis due to hypoxia. Increase in the transcripts of citrate synthase correlates with the increased protein expression supporting increased mitochondrial activity. Interestingly, the PGC1-α transcript level was higher while the expression of PGC1-α protein was downregulated in hypoxic cells compared to normoxic control. PGC1-α upregulates BAX expression and downregulates Bcl2 in several cell types revealing its role in inducing apoptosis by increasing BAX:Bcl2 ratio. The increased BAX:Bcl2 ratio destabilizes the mitochondrial membrane which results in the release of cytochrome c to the cytosol which in turn triggers apoptosis^40–42^. These reports correlate with our findings from the RCI rats in which the ratio decreased drastically during the course of healing. The elevated BAX:Bcl2 ratio under extreme hypoxia may elicit an inhibitory effect on PGC1-α resulting in its downregulation at the protein level, which warrants further research. None-the-less, the tenocytes respond to hypoxia and associated complications by increasing the mitochondrial number.

The integrity of mitochondrial membrane is critical to prevent the leakage of pro-apoptotic mediators from mitochondria^43^. The mitochondrial membrane pore transition reflects the health status of mitochondria since it results in membrane depolarization, inhibition of ATP synthesis, Ca^2+^ overload, matrix swelling, and the release of pro-apoptotic mediators^44^. The reduction of calcein fluorescence in the hypoxic tenocytes revealed the loss of the integrity of mitochondrial membrane pores. However, the fluorescence signal for mitochondria (MitoTracker) was found to be higher in hypoxic tenocytes when compared to normoxic cells. These findings suggest that the tenocytes under hypoxia undergo mitochondrial dysfunction, and mitochondrial number increases to retain the metabolic demand of the cells and thus inducing an overall positive effect. Also, this correlated with the upregulation of mitochondrial biomarkers following RCI in our experimental rats. Moreover, the heterodimeric cytoskeletal protein, tubulin (composed of α and β sub-units) interacts with mitochondrial membrane and associates it with the membranes of other organelles. Tubulin exhibits a strong affinity with mitochondrial outer membrane, especially to voltage-dependent anion channels^45^. In addition, the level of tubulin in the cells represents mitochondrial density as evident from the reports using anti-tubulin agents^43^. The hypoxic tenocytes in our study exhibited an upregulation of β-tubulin which represents an increase in mitochondrial density.

Generally, the tenocytes are characterized with very few numbers of mitochondria under normal conditions^46^. Interestingly, we observed an increase in the number of mitochondria in our hypoxic tenocytes when compared with normoxic cells as examined by the qualitative TEM analysis. However, no considerable difference in the mitochondrial aspect ratio was observed between hypoxic and normoxic tenocytes. Moreover, the pathological features including coordination of the trans-mitochondrial cristae, extensive mitochondrial compartmentalization and the extension of outer mitochondrial membrane were evident in hypoxic tenocytes^47,48^. These findings suggest that hypoxia triggers mitochondrial biogenesis in tenocytes to compromise the energy requirements and to replenish the raw materials for cell recovery, however exhibits alterations in their characteristics^33,49^.

Our study has few limitations. The limited amount of rat shoulder tendon tissue did not allow us to perform mRNA and TEM analysis for mitochondria and created difficulty to identify muscle-tendon interface after RCI. In addition, presence of suture in the RC tendon caused the loss of tissue during processing and staining. Finally, we used young age rats that have active healing responses. Further research is warranted on cytokine signaling and inflammation with and without hypoxia that could influence mitochondrial pathology in tenocytes. The mitochondrial physiology and pathology following hypoxic stress associated with RCI could be due to the combinatorial effects of other factors such as inflammation, cytokine signaling, DAMPs and the availability of pro-survival proteins which were difficult to simulate *in vitro*. Nonetheless, the findings in the present study revealed an association of hypoxia and mitochondrial activity following RCI where the increase in mitochondrial density could facilitate the healing response. The strategies which activate mitochondrial biogenesis could have translational significance in the management of RCI.

## Materials and Methods

### Animals

Institutional Animal Care and Use Committee of Creighton University approved the experimental animal research protocol. All methods in the animal care and procedures in this research protocol were performed in accordance with the NIH and OLAW guidelines. Power analysis was carried out to determine the effective population size for the animal studies using G*Power 3.0.1.0 software and A Priori power analysis tool by fixing alpha error to be 0.05. The effective size was chosen to be 0.35–0.5 based on the expected difference between the RCI and control groups. The primary outcome measure is the decrease in lesion size. In order to attain significant RCI with respect to control, the sample size necessary to have at least 80% power will be 7 in each experimental group. Sprague Dawley rats (male) (Charles River Laboratories, USA) of age 8–10 weeks and weighting 230–250 g were purchased for the studies. The animals were given food and drinking water *ad libitum* with 12/12 light-dark cycle throughout the study.

### Shoulder tenotomy, tissue harvest and processing

The study consisted of 3 groups, with 7 rats in each group – Group-A; 3–5 days post-injury, Group-B; 10–12 days post-injury and Group-C; 22–24 days post-injury. Sterile conditions were maintained during the surgical procedures. Hairs around the shoulder were removed, the skin was disinfected and wiped with ethanol prior to surgery. The animals were then anesthetized with 80 mg/kg ketamine (IP) and 5 mg/kg xylazine (IM) and anesthesia was maintained with 2% isoflurane^50^. The animals were then placed in the right lateral position and the shoulder region was covered with sterile drapes. In the lateral aspect of the shoulder over the acromion, a skin incision was made and the deltoid muscle was sharply detached from the postero-lateral aspect of the acromion which was followed by the retraction of the acromion. A suture was placed through supraspinatus and infraspinatus at the musculo-tendon junction to control the tendon stumps after detachment. Then supraspinatus and infraspinatus tendons were detached from the greater tuberosity using a No. 11 surgical blade and the tendon stump from lateral side was resected further in order to prevent the healing of the tendon stump back to the greater tuberosity. The skin incision was then closed using sterile metallic skin staples. The animals recovered from anesthesia after around 45 min and were then brought back to normal cages till the sacrifice. Buprenorphine 0.01 mg/Kg was given subcutaneously twice a day for post-operative pain management followed by the oral administration of 5 mg acetaminophen each day for another 5 days. Post-operative pain assessment and examination of the cage activities were conducted regularly for 10 days post-surgery. The Group-A, Group-B and Group-C rats were sacrificed post-surgery after 3–5 days, 10–12 days and 22–24 days, respectively.

After sacrifice the shoulder tendon from each animal was collected, fixed in formalin at room temperature for 24 h, embedded in paraffin wax and sections of 5 μm thickness were taken onto microscopic slides for immunofluorescence analysis.

### Immunofluorescence

The protein expression of biomarkers to assess mitochondrial status in the tendon tissue sections was analyzed by immunostaining following our previously reported protocols^29,38^. Briefly, the sections were subjected to antigen retrieval by heating at 95 °C for 20 min in HIER buffer (Heat Induced Antigen Retrieval) (TA-135-HBM) followed by the use of blocking solution (0.25% Triton X-100 and 5% horse serum in PBS) at room temperature for 2 hrs. Primary antibodies against Bcl2 (sc-7382, Santa Cruz Biotechnology, Inc), BAX (ab-32503, Abcam), citrate synthase (ab-96600, Abcam), complex-1 and PGC-1α (sc-518025, Santa Cruz Biotechnology, Inc) were used in a dilution of 1:50. Corresponding fluorochrome-conjugated secondary antibodies with a dilution of 1:200 were used to bind the primary antibodies. Nuclei were counterstained with 4′,6-diamidino-2-phenylindole (DAPI) (H-1200) and imaged using a fluorescent microscope (Olympus BX51; Olympus America, Center Valley, PA). The fluorescence intensity was quantified using ImageJ software and the results are expressed as mean fluorescence intensity (MFI). A negative control with secondary antibodies alone was maintained in a similar manner to detect background fluorescence and to fix the exposure time.

### Isolation of tenocytes from swine shoulder tendon and maintenance

The skin around the shoulder region of the Yucatan microswine (Sinclair Research) (female, 6–8 months old) was cleaned twice using iodine solution and then with 70% isopropanol. A skin incision was made at the shoulder region using a sterile scalpel blade (#20) and infraspinatus tendon tissue was harvested. The tissue was then washed in serum free DMEM (D-6429, Sigma Aldrich) containing antibiotics, minced to very fine pieces and digested with collagenase-1 at 37 °C with intermittent shaking for 2 hours. The mixture was then centrifuged at 200 g for 5 min to settle down the undigested tissue. The partially digested tendon tissue was placed to culture flask for explant culture. The tendon cells started to migrate out of the tissue pieces and started colonizing after 5–6 days of initial seeding and became confluent after two weeks. DMEM containing 20% FBS and antibiotics were used for the isolation and maintenance of the cells. The culture was maintained at 37 °C and 5% CO_2_ in a humidified incubator and after 6 days the unattached cells were removed and replenished with fresh DMEM. The cells were isolated from four micro swine, pooled (passage 2–4) and stored under liquid nitrogen. The cell culture experiments were conducted in quadruplicate by reviving the cells for each experimental repeat. Collagen-1-coated (Zen-Bio) culture wares were used for culturing the cells.

### Characterization of swine tenocytes

The cells (passage 2) were grown in a chamber slide to 70% confluence in DMEM. The media was then removed, washed in sterile PBS, immediately fixed with 5% formalin for 30 min and immunofluorescence assay for tendon specific biomarkers including tenomodulin and scleraxis were carried out by following the above-mentioned protocol. The primary antibodies used were anti-goat tenomodulin (sc-49324, Santa Cruz Biotechnology, Inc) and anti-goat scleraxis (sc-87425, Santa Cruz Biotechnology, Inc), and the secondary antibody used was donkey-anti-goat-594. The images were acquired using a fluorescent slide scanner system (VS120-S6-W, Olympus) at 20x magnification and the images were taken and converted to TIF format using OlyVIA Desktop software. DAPI was used to counterstain the nuclei.

### Protein expression of mitochondrial markers under hypoxia

The tenocytes cultures were incubated 20–24 h in a hypoxia chamber by passing N_2_ gas to maintain pO_2_ at 2%. After hypoxia treatment, the cells were immediately fixed with 10% formalin for 20 min and immunofluorescence of mitochondrial markers such as complex-1, citrate synthase, apoptosis regulators including BAX and Bcl2 and β-tubulin (ab-18207, Abcam) were conducted. The cells grown under normoxic conditions served as control and the experiments were done in triplicates. The cells (n = 30) were individually analyzed for MFI determination using the polygon selection tool of ImageJ software.

### qRT-PCR

Total cellular RNA was isolated by trizol method from hypoxic and normoxic tenocytes and 1 μg RNA from each group was reverse transcribed to cDNA using cDNA synthesis kit (Promega) following the manufacturer’s protocol. The reaction mixture was prepared for 40 μl reaction volume. The mRNA transcripts for the genes PGC1-α, citrate synthase, BAX, and Bcl2 were amplified and quantified by real-time PCR (Applied Biosystems, CA, U.S.A.) using SYBR Green chemistry using corresponding forward and reverse primers for each gene (Table 2). The 18s rRNA was used as housekeeping reference gene. The program set up was 95 °C for 10 minutes; and 40 cycles of 95 °C for 15 s, 60 °C for 1 min. The mRNA expression of the genes was normalized with the expression level of 18s rRNA. The forward and reverse primers for the PCR amplification were designed using NCBI Primer-BLAST tool. The melt-curve analysis was performed to confirm the absence of non-specific amplification products and primer dimers. The fold-change of mRNA expression of the mitochondrial markers was determined by 2^−ΔΔCT^ method using 18s rRNA as a housekeeping gene and tenocytes grown under normoxia were used as control. The experiments were conducted in quadruplicates and the results are represented as fold-change with respect to normoxic control^29^.

**Table 2:** Primers used for RT-PCR analysis.

Bcl2		Transcript id
Fw	5′TGTGTGGAGAGCGTCAACCG3′	XR_002346028.1
Rw	5′CAGCCCACCCACACTCCAA3′	XR_002346028.1
**BAX**		**Transcript id**
Fw	5′GATGGACGGGTCCGGGGAG3′	NC_010448.4
Rw	5′GCCCAGCTCAGGTGTCTCTC3′	NC_010448.4
PGC1α		**Transcript id**
Fw	5′ACATGTGCAACCAGGACTCTGT3′	NC_010450.4
Rw	5′ACTGCACCACTTGAGTCCACC3′	NC_010450.4
**Citrate synthase**		**Transcript id**
Fw	5′CCTGCCATGGCCTTACTCACT3′	NM_214276.1
Rw	5′CGGGCAGCAAGAACAAGACA3′	NM_214276.1
**18s rRNA**		**Transcript id**
Fw	5′ACGTTGGCGAGAGCGTGG3′	XM_021081891.1
Rw	5′AGGTGGAGGAGGCGAGAGAG3′	XM_021081891.1

### Determination of mitochondrial superoxide

The status of ROS and oxidative stress in the tenocytes cultured under hypoxia was examined by determining mitochondrial superoxide using MitoSOX Red (M36008, Invitrogen). MitoSOX Red selectively targets mitochondria and undergoes oxidation by superoxide to form a stable fluorescent compound which was imaged using the green filter of fluorescent microscope. The tenocytes were grown under hypoxia in 4 well chamber slides and were incubated with 5 μM MitoSOX Red reagent (Invitrogen) for 10 min following the manufacturer’s instructions^51^. Then, the live cell imaging was performed using a fluorescent slide scanner (VS120-S6-W, Olympus) after mounting with DMEM (serum free). The cells grown under normoxia were used as the control. The experiments were run in quadruplicate and the MFI values were normalized to 100 cells and were compared with the control.

### Mitochondrial pore transition assay

The mitochondrial health of tenocytes grown under hypoxic conditions was determined by the assessment of mitochondrial pore permeability using Image-iT LIVE mitochondrial transition pore assay Kit (I35103, Invitrogen). The experiment was conducted by following the protocol provided by the manufacturer with minor modifications. Briefly, the mitochondrial membrane is permeable to acetoxymethyl ester of calcein dye (Calcein-AM) and intra-cellular esterases convert Calcein-AM to calcein, which is a fluorescent impermeable compound. The CoCl_2_ quenches cytosolic calcein and the healthy mitochondrial membrane is impermeable CoCl_2_ resulting in the retention of mitochondrial calcein fluorescence. However, the activation of mitochondrial pores using ionophores cause the overload of mitochondrial calcium which triggers the pore activation and the uptake of CoCl_2_ by mitochondria, resulting in quenching of fluorescence. MitoTracker Red was used to label mitochondria. The tenocytes grown in chamber slides under hypoxia were washed with serum free DMEM and incubated in 200 µl labeling solution (1 µl each of 1 mM calcein AM, 200 µM MitoTracker Red, 1 mM Hoechst 33342, and 1M CoCl_2_ in 1 ml serum free DMEM) at 37 °C for 15 min. After incubation, the cells were washed with serum free DMEM and immediately imaged under fluorescence slide scanner (VS120-S6-W, Olympus)^52^. The cells grown under normoxic conditions were also treated in a similar manner for comparison. The experiments were run in quadruplicate and the MFI values were normalized to 100 cells and were compared with the normoxic control.

### Mitochondrial morphology by transmission electron microscopy (TEM)

The hypoxic and normoxic swine tenocytes were trypsinized, and centrifuged at 350 g for 5 min to form the pellet. The pellet was then fixed overnight using 2% glutaraldehyde in 0.1 M Sorenson’s buffer (pH 7.4) at 4 °C and ultra-thin sections of the cell pellets were made using ultra-microtome for TEM analysis following the previously published protocols^47,48^. The sections for TEM imaging were spotted onto formvar/silicon monoxide coated 200 mesh copper grids (Ted Pella Inc. Redding, CA). Grids were glow discharged for 60 seconds at 20 µA with a GloQube glow discharge unit (Quorum Technologies, East Sussex, UK) prior to use. The sections were negatively stained with NanoVan (Nanoprobes, New York, NY) and examined on a Tecnai G^2^ Spirit TWIN (FEI, Hillsboro, OR) operating at an accelerating voltage of 80 kV. Images were acquired digitally with an AMT (Woburn, MA) digital imaging system. The images were assessed for the morphological features such as paracrystalline inclusions, cristae organization, concentric layering of cristae membrane, matrix compartmentalization, and nano-tunneling to examine the mitochondrial pathology^47^. The aspect ratio (ratio of length to width) was calculated from the linear axis and cross-sectional axis of mitochondria using ImageJ software. The incomplete images of mitochondria were omitted from the analysis.

### Statistical analysis

The results of immunofluorescence intensity in the rat tendon tissues (n = 7) and cell culture experiments (n = 4) were expressed as mean ± SEM. The statistical significance for the rat tendon tissues was evaluated by two-way ANOVA with Tukey’s multiple comparison test using GraphPad Prism software. The images were randomly taken from different fields in each tendon specimen for MFI quantification. 1–2 sections from controls and 2–3 sections from experimental groups were analyzed depending on the tissue size and the adherence of tissue section to the microscopic slides. Some thin sections of the tissue from experimental groups did not adhere to the slides due to the presence of sutures. The intensity of each tissue specimen corresponds to the average MFI of 2–4 images acquired randomly from different fields. The average values of all individual specimens from each group were utilized to calculate average mean and SEM to perform statistical analysis and to compare with the contralateral controls in the same experimental group. The MFI for the mitochondrial biomarkers and mitochondrial pore transition (MPT) from the cell culture experiments were determined by the random analysis of 30 cells from four experimental repeats. The statistical significance for the *in vitro* experiments was determined by ANOVA followed by Tukey’s multiple comparisons test using GraphPad Prism software. Unpaired ‘*t*’ test was employed to compare the effect of hypoxia using MitoSox and mitochondrial aspect ratio as measured by TEM. The *p* < *0.05* values were considered to be significant in all experiments.

## Conclusion

In the surgically-induced tenotomy model of RCI rats, there was an upregulation of mitochondrial (Complex-1, citrate synthase) and apoptotic biomarkers (BAX, Bcl2) in the early phase of tendon healing which were found to be decreased as the healing proceeded. A similar response of these mediators was evident in cultured swine tenocytes under hypoxic conditions. The tenocytes grown under hypoxic conditions exhibited increased superoxide content and possessed alterations in mitochondrial membrane pore integrity. An increase in the number of mitochondria in tenocytes following the hypoxic treatment suggest their critical role in sustaining the energy metabolism. The findings in this study support the association of hypoxia and mitochondrial activity following RCI where the increase in mitochondrial density drives the healing responses. The strategies to activate mitochondrial biogenesis may have greater translational significance in the management of RCI.

## Electronic supplementary material

Supplementary Table 1
