# Supplementary material for: Amplification of Mitochondrial Activity in the Healing Response Following Rotator Cuff Tendon Injury
Source: Sci Rep. 2018 Nov 19;8:17027. doi: 10.1038/s41598-018-35391-7 (PMC6242817; doi:10.1038/s41598-018-35391-7)
Supplement: Supplementary file 1 — Supplementary Table 1 [file 41598_2018_35391_MOESM1_ESM.docx]

**Supplementary Information**

**Manuscript #:** SREP-18-22495C

**Manuscript Title:** Amplification of Mitochondrial Activity in the Healing Response Following Rotator Cuff Tendon Injury

**Authors:** Finosh G Thankam, Isaiah S Chandra, Anuradha N Kovilam, Connor G Diaz, Benjamin T Volberding, Matthew F Dilisio, Mohamed M Radwan, R. Michael Gross and Devendra K. Agrawal

Departments of Clinical & Translational Science and Orthopedic Surgery, Creighton University School of Medicine, Omaha, NE 68178, USA

| **Citrate Synthase** | | |
| --- | --- | --- |
| **Comparison** | **Significance** | **P Value** |
| Group A:Control vs. Group A:RCI | **** | < 0.0001 |
| Group A:Control vs. Group B:Control | ns | 0.9777 |
| Group A:Control vs. Group B:RCI | **** | < 0.0001 |
| Group A:Control vs. Group C:Control | ns | 0.9829 |
| Group A:Control vs. Group C:RCI | ns | 0.0528 |
| Group A:RCI vs. Group B:Control | **** | < 0.0001 |
| Group A:RCI vs. Group B:RCI | ns | 0.9952 |
| Group A:RCI vs. Group C:Control | **** | < 0.0001 |
| Group A:RCI vs. Group C:RCI | * | 0.0469 |
| Group B:Control vs. Group B:RCI | **** | < 0.0001 |
| Group B:Control vs. Group C:Control | ns | > 0.9999 |
| Group B:Control vs. Group C:RCI | * | 0.0106 |
| Group B:RCI vs. Group C:Control | **** | < 0.0001 |
| Group B:RCI vs. Group C:RCI | ns | 0.1583 |
| Group C:Control vs. Group C:RCI | * | 0.0201 |
| **Complex 1** | | |
| **Comparison** | **Significance** | **P value** |
| Group A:Control vs. Group A:RCI | *** | 0.0008 |
| Group A:Control vs. Group B:Control | ns | 0.9901 |
| Group A:Control vs. Group B:RCI | **** | < 0.0001 |
| Group A:Control vs. Group C:Control | ns | 0.9854 |
| Group A:Control vs. Group C:RCI | ns | 0.9857 |
| Group A:RCI vs. Group B:Control | *** | 0.0002 |
| Group A:RCI vs. Group B:RCI | ns | 0.2649 |
| Group A:RCI vs. Group C:Control | *** | 0.0001 |
| Group A:RCI vs. Group C:RCI | ** | 0.0075 |
| Group B:Control vs. Group B:RCI | **** | < 0.0001 |
| Group B:Control vs. Group C:Control | ns | > 0.9999 |
| Group B:Control vs. Group C:RCI | ns | 0.8289 |
| Group B:RCI vs. Group C:Control | **** | < 0.0001 |
| Group B:RCI vs. Group C:RCI | **** | < 0.0001 |
| Group C:Control vs. Group C:RCI | ns | 0.7915 |
| **BAX** | | |
| **Comparison** | **Significance** | **P Value** |
| Group A:Control vs. Group A:RCI | **** | < 0.0001 |
| Group A:Control vs. Group B:Control | ns | > 0.9999 |
| Group A:Control vs. Group B:RCI | **** | < 0.0001 |
| Group A:Control vs. Group C:Control | ns | 0.9997 |
| Group A:Control vs. Group C:RCI | ns | 0.1312 |
| Group A:RCI vs. Group B:Control | **** | < 0.0001 |
| Group A:RCI vs. Group B:RCI | ns | 0.2556 |
| Group A:RCI vs. Group C:Control | **** | < 0.0001 |
| Group A:RCI vs. Group C:RCI | **** | < 0.0001 |
| Group B:Control vs. Group B:RCI | **** | < 0.0001 |
| Group B:Control vs. Group C:Control | ns | > 0.9999 |
| Group B:Control vs. Group C:RCI | ns | 0.083 |
| Group B:RCI vs. Group C:Control | **** | < 0.0001 |
| Group B:RCI vs. Group C:RCI | ** | 0.0056 |
| Group C:Control vs. Group C:RCI | ns | 0.0704 |
| **Bcl2** | |  |
| **Comparison** | **Significance** |  |
| Group A:Control vs. Group A:RCI | *** | 0.0001 |
| Group A:Control vs. Group B:Control | ns | > 0.9999 |
| Group A:Control vs. Group B:RCI | * | 0.0321 |
| Group A:Control vs. Group C:Control | ns | > 0.9999 |
| Group A:Control vs. Group C:RCI | ** | 0.0029 |
| Group A:RCI vs. Group B:Control | **** | < 0.0001 |
| Group A:RCI vs. Group B:RCI | ns | 0.5942 |
| Group A:RCI vs. Group C:Control | **** | < 0.0001 |
| Group A:RCI vs. Group C:RCI | ns | 0.943 |
| Group B:Control vs. Group B:RCI | * | 0.0164 |
| Group B:Control vs. Group C:Control | ns | > 0.9999 |
| Group B:Control vs. Group C:RCI | ** | 0.0012 |
| Group B:RCI vs. Group C:Control | * | 0.0165 |
| Group B:RCI vs. Group C:RCI | ns | 0.9762 |
| Group C:Control vs. Group C:RCI | ** | 0.0012 |

**Supplementary Table 1**: Statistical analysis and multiple comparison of the expression of mitochondrial biomarkers in the rat tendon tissues using two-way ANOVA. The *p<0.05* values were considered to be significant in all experiments. (**** P<0.0001; *** P<0.001; ** P<0.01; * P<0.05; ns – non-significant, P>0.05)
